# Inflamação e Fibrilação Atrial: Uma Associação Exclusiva ou Cúmplice do Continuum Cardiovascular de Fatores de Risco Adicionais?

**DOI:** 10.36660/abc.20240382

**Published:** 2024-08-01

**Authors:** Francisco Darrieux

**Affiliations:** 1 Hospital das Clínicas da Faculdade de Medicina da Universidade de São Paulo Instituto do Coração Unidade de Arritmias Cardíacas São Paulo SP Brasil Unidade de Arritmias Cardíacas – Instituto do Coração do Hospital das Clínicas da Faculdade de Medicina da Universidade de São Paulo, São Paulo, SP – Brasil

**Keywords:** Fibrilação Atrial, Inflamação, Mecanismos

O papel da inflamação sistêmica na promoção de doenças cardiovasculares tem atraído a atenção de diversos pesquisadores.^1^ A inflamação pode ser protetora, pois também faz parte da resposta à infecção e à lesão, desempenhando um papel nos processos de defesa e cura.^2^ No entanto, a resposta inflamatória pode persistir além da ameaça original, levando à inflamação crônica, remodelação tecidual adversa e doença.^1,2^

A relação inflamação na ocorrência de FA tem sido um problema recorrente há mais de 20 anos.^3,4^ Há evidências crescentes que sugerem uma ligação direta entre a inflamação sistêmica ou local e o desenvolvimento de FA. Condições inflamatórias cardíacas locais, incluindo pericardite e miocardite, também aumentam a incidência de FA,^5^ bem como casos graves de doença por coronavírus.^6^ Alguns estudos demonstraram ligação entre biomarcadores inflamatórios e FA, mas uma das limitações desses estudos é que geralmente utilizam um único biomarcador, resultando em achados heterogêneos.^6,7^

O estudo de Naser et al.^8^ tenta explorar a associação entre alguns parâmetros relacionados à carga da inflamação e a carga da FA, gerando reflexões e hipóteses de trabalho. O papel dos marcadores de inflamação e a sua associação com o perfil dos pacientes com FA é muito enriquecedor, dependendo da carga e da temporalidade da FA. O estudo utilizou o índice de imunoinflamação sistêmica, abreviado como SII ( SII=neutrófilos×plaquetas/linfócitos ), sendo um dos marcadores que demonstraram predizer o estado inflamatório sob diversas condições em estudos anteriores. Foi uma análise transversal de 453 pacientes com FA (138 FA paroxística e 315 FA permanente). O papel preditivo do SII e de outros marcadores inflamatórios na probabilidade do padrão de FA foi avaliado por análises de regressão logística. Idade, pressão arterial diastólica, frequência cardíaca, diabetes mellitus (DM), neutrófilos, relação plaquetas-linfócitos (RPL), relação neutrófilos-linfócitos (RNL), SII, proteína C reativa (PCR), largura de distribuição de glóbulos vermelhos (RDW), hemoglobina A1-C (HBA1C) e diâmetro do átrio esquerdo (DAE) foram significativamente maiores no grupo com FA permanente. Após análise de regressão logística, idade (p=0,038), DM (p=0,024), RDW (p=0,023), PCR (p=0,010), SII (p=0,001) e DAE (p<0,001) contribuíram significativamente para a previsão da probabilidade de FA permanente. Concluíram que o SII estava independentemente associado à carga de FA.

Parcialmente em linha com os achados de Naser et al.,^8^ uma publicação recente derivada da coorte UK Biobank avaliou de forma abrangente a associação entre vários indicadores de inflamação sistêmica e FA, arritmia ventricular (AV) e bradiarritmia.^9^ Um total de 478.524 indivíduos elegíveis foram incluídos e 24.484 eventos incidentes de FA ocorreram em 5,88 milhões de pessoas-ano de acompanhamento (taxa de incidência: 4,16 eventos por 1.000 pessoas-ano, IC de 95%: 4,11–4,21). Após ajuste para todas as possíveis variáveis de confusão, os níveis de PCR foram significativamente associados ao risco de FA. Eles também descobriram que a FC para FA incidente aumentou significativamente com um aumento na contagem de neutrófilos, contagem de monócitos e RNL. Também foi observada uma relação em forma de U entre SII e risco de FA, mesmo após ajuste completo das covariáveis. Todas essas tendências e associações com arritmias, incluindo FA, foram validadas em diferentes populações através de análises de subgrupos e análises de sensibilidade.^9^

O estudo de Naser et al.^8^ possui bons dados para serem interpretados, porém, por ser um desenho de análise transversal, pode ter gerado hipóteses tendenciosas na interpretação da causalidade. Falta um grupo controle sem FA e com as mesmas comorbidades. Contudo, esta questão (falta de um grupo de controle) foi levantada no parágrafo das limitações.

Outra questão a ser levantada: os marcadores de inflamação são realmente preditores ou seus achados estão associados ao processo cardiovascular contínuo da FA? Esta questão deve ser levantada com cautela e são necessários estudos prospectivos para determinar se o SII pode ser útil na identificação de pacientes com alto risco de progressão da FA.

As razões pelas quais a FA foi considerada permanente também são desafiadoras. Alguns pacientes com FA podem ser submetidos a procedimentos de ablação (um ou mais) e os dados demográficos do estudo mostraram que esta intervenção ocorreu em apenas 12,8% dos casos.^8^ A DAE também foi um preditor independente de FA permanente. Sabe-se também que, como a FA pode progredir para formas persistentes/permanentes (independentemente da ablação por cateter da FA ou de falhas na terapia médica), a probabilidade de aumento do AE é esperada. Por outro lado, pacientes submetidos à ablação por cateter de FA com sucesso podem apresentar remodelamento do AE.^10^ Vários estudos de metanálise demonstraram reduções significativas na DAE e nos volumes no acompanhamento pós-ablação, especialmente naqueles sem recorrências de FA.^11,12^ Esses pacientes poderiam ter apresentado marcadores inflamatórios mais elevados antes, que foram reduzidos após a intervenção e manutenção do ritmo sinusal? Estes dados podem ser interpretados com cautela, uma vez que a DAE pode não ser um preditor independente de formas de FA permanente, uma vez que parte destas questões pode ser explicada pelas estratégias de tratamento que foram adotadas durante a jornada do paciente com FA.

Além disso, outras fronteiras de conhecimento e perfis de dados exploratórios tentam descobrir outras vias de sinalização inflamatória, além da visão clássica de que a inflamação resulta da produção de citocinas por uma variedade de glóbulos brancos infiltrantes em resposta a lesões teciduais e/ou respostas de células imunes. Começaram a surgir algumas evidências de que outros tipos de células, incluindo os cardiomiócitos, podem ter efeitos inflamatórios potencialmente importantes.^13^ Os cardiomiócitos atriais possuem componentes da maquinaria de sinalização inflamatória do inflamassoma NLRP3, que é ativado em modelos animais e pacientes com FA. Os estudos desses modelos animais sugerem que a ativação do inflamassoma NLRP3 em cardiomiócitos atriais pode ser uma condição para a ocorrência de FA.^13^

Uma questão que naturalmente vem à mente é: qual a aplicabilidade clínica dos sinais inflamatórios e a geração de novos medicamentos com terapias-alvo específicas para essas vias de sinalização pró-inflamatórias e geradoras de FA? Curiosamente, parte do arsenal terapêutico que visa reduzir a atividade inflamatória não tem efeito na redução do risco de FA, como inibidores de COX, aspirina e glicocorticoides, podendo até aumentar outros riscos, dependendo do medicamento, como sangramento, elevação do nível sanguíneo níveis pressóricos ou protrombose.^13^ O estudo COP-AF232^14^ infelizmente demonstrou que em pacientes submetidos a cirurgia torácica não cardíaca de grande porte, a administração de colchicina não reduziu significativamente a incidência de FA clinicamente importante, mas aumentou o risco de diarreia não infecciosa, principalmente benigna. Portanto, a intervenção "anti-inflamatória" na prevenção da FA incidental ainda se torna um desafio, uma vez que os seus mecanismos ainda não estão totalmente estabelecidos. Além disso, a maioria dos pacientes com FA apresenta fatores de risco adicionais relacionados ao aumento da atividade inflamatória, como obesidade, apneia do sono, insuficiência cardíaca e diabetes mellitus.

Portanto, a fisiopatologia das arritmias é complexa e a inflamação provavelmente desempenha um papel tanto no início quanto na duração dos episódios. Contudo, o papel da farmacoterapia voltada para a inflamação e prevenção de arritmias permanece obscuro, provavelmente porque a FA é quase sempre uma doença multifatorial e porque o alvo estudado na prevenção desta carga inflamatória também pode estar presente nas comorbidades da FA.
