# Supplementary material for: Inflamação e Fibrilação Atrial: Uma Associação Exclusiva ou Cúmplice do Continuum Cardiovascular de Fatores de Risco Adicionais?
Source: Arq Bras Cardiol. 2024 Aug 1;121(6):e20240382. [Article in Portuguese] doi: 10.36660/abc.20240382 (PMC11364447; doi:10.36660/abc.20240382)
Supplement: Translation

# Translation: Inflammation and Atrial Fibrillation: An Exclusive Association or an Accomplice to the Cardiovascular Continuum of Additional Risk Factors?

**DOI:** 10.36660/abc.20240382i

**Authors:** Francisco Darrieux

**Affiliations:** 1 São Paulo SP Brazil Unidade de Unidade de Arritmias Cardíacas – Instituto do Coração do Hospital das Clínicas da Faculdade de Medicina da Universidade de São Paulo, São Paulo, SP – Brazil

**Keywords:** Atrial Fibrillation, Inflammation, Mechanism

The role of systemic inflammation in promoting cardiovascular diseases has attracted the attention of several researchers.^1^ Inflammation can be protective as also being part of the response to infection and injury, playing a role in the defense and healing processes.^2^ However, the inflammatory response can persist beyond the original threat leading to chronic inflammation, adverse tissue remodeling, and disease.^1,2^

The inflammation relationship in the occurrence of AF has been a recurring issue for more than 20 years.^3,4^ There is increasing evidence suggesting a direct link between systemic or local inflammation and the development of AF. Local cardiac inflammatory conditions, including pericarditis and myocarditis, also increase the incidence of AF,^5^ as well as severe coronavirus disease cases.^6^ Some studies have demonstrated a link between inflammatory biomarkers and AF, but one of the limitations of these studies is that generally use a single biomarker, resulting in heterogenous findings.^6,7^

The study by Naser et al.^8^ attempts to explore the association between some parameters related to the burden of inflammation with the burden of AF, generating reflections and working hypotheses. The role of inflammation markers and their association with the profile of patients with AF is very enriching, depending on the burden and temporality of the AF. The study used the systemic immunoinflammation index, abbreviated as SII ( SII=neutrophils×platelets/lymphocytes ), being one of the markers that have been shown to predict inflammatory status under various conditions in previous studies [18–20]. It was a cross-sectional analysis of 453 patients with AF (138 paroxysmal AF and 315 permanent AF). The prediction role of SII and other inflammatory markers in the likelihood of AF pattern was evaluated by logistic regression analyses. Age, diastolic blood pressure, heart rate, diabetes mellitus (DM), neutrophil, platelet-lymphocyte ratio (PLR), neutrophil-lymphocyte ratio (NLR), SII, C- reactive protein (CRP), red blood cell distribution width (RDW), hemoglobin A1-C (HBA1C) and left atrial diameter (LAD) were significantly higher in permanent AF group. After logistic regression analysis, age (p=0.038), DM (p=0.024), RDW (p=0.023), CRP (p=0.010), SII (p=0.001), and LAD (p<0.001) significantly contributed to the prediction of the likelihood of permanent AF. They concluded that SII was independently associated with the AF burden.

Partially in line with the findings of Naser et al.,^8^ a recent publication derived from the UK Biobank cohort comprehensively assessed the association between several indicators of systemic inflammation and AF, ventricular arrhythmia (VA), and bradyarrhythmia.^9^ A total of 478,524 eligible individuals were included and 24,484 incident AF events occurred in 5.88 million person-years of follow-up (incidence rate: 4.16 events per 1,000 person-years, 95% CI: 4.11–4.21). After adjusting for all possible confounding variables, CRP levels were significantly associated with the risk of AF. They also found that the HR for incident AF increased significantly with an increase in neutrophil count, monocyte count, and NLR. A U-shaped relationship between SII and AF risk was also observed, even after full covariate adjustment. All these trends and associations with arrhythmias, including AF, were validated in different populations through subgroup analyses and sensitivity analyses.^9^

The study by Naser et al.^8^ has good data to be interpreted, however, as a cross-sectional analysis design, it may have generated biased hypotheses in the interpretation of causality. A control group without AF and with the same comorbidities is missing. However, this issue (lack of a control group) was raised in the limitations paragraph.

Another question to be raised: are inflammation markers really predictors or are their findings associated with the *continuum* cardiovascular process of AF? This question must be raised with caution and prospective studies are needed to determine whether SII may be useful in identifying patients at high risk for AF progression.

The reasons why AF was considered permanent are also challenging. Some patients with AF can be submitted to ablation procedures (one or more) and the demographic data of the study showed that this intervention occurred in only 12,8% of the cases.^8^ LAD was also an independent predictor of permanent AF. It is also known that as AF can progress to persistent/permanent forms (regardless of AF catheter ablation or failures in medical therapy), the likelihood of LA enlargement is expected. On the other hand, patients who undergo successful AF catheter ablation may experience LA remodeling.^10^ Several meta-analysis studies have demonstrated significant reductions in LAD and volumes in post-ablation follow-up, especially in those without AF recurrences.^11,12^ Could these patients have presented higher inflammation markers before, which were reduced after the intervention and maintenance of sinus rhythm? These data might be interpreted with caution, as LAD may not be an independent predictor of permanent AF forms, as part of these issues may be explained by the treatment strategies that were adopted during the AF patient's journey.

Furthermore, other frontiers of knowledge and exploratory data profiling attempt to discover other inflammatory signaling pathways, beyond the classic view that inflammation results from the production of cytokines by a variety of infiltrating white blood cells in response to tissue injury and/or immune cell responses. Some evidence has begun to emerge that other cell types, including cardiomyocytes, may have potentially important inflammatory effects.^13^ Atrial cardiomyocytes have inflammatory signaling machinery components of the NLRP3 inflammasome, which is activated in animal models and patients with AF. These animal models’ studies suggest that activation of the NLRP3 inflammasome in atrial cardiomyocytes may be a condition for the occurrence of AF.^13^

A question that naturally comes to mind is: what is the clinical applicability of inflammatory signals and the generation of new medications with specific target therapies for these pro-inflammatory and AF-generating signaling pathways? Intriguingly, part of the therapeutic arsenal aimed at reducing inflammatory activity has no effect on reducing the risk of AF, such as COX inhibitors, aspirin, and glucocorticoids, and may even increase other risks, depending on the drug, such as bleeding, elevated blood pressure levels or prothrombosis.^13^ The COP-AF232 study^14^ has unfortunately demonstrated that in patients undergoing major non-cardiac thoracic surgery, administration of colchicine did not significantly reduce the incidence of clinically important AF but increased the risk of non-infectious diarrhea, mostly benign. Therefore, "anti-inflammatory" intervention in the prevention of incidental AF still becomes challenging since its mechanisms are not yet fully established. Furthermore, most patients with AF have additional risk factors that are related to increased inflammatory activity, such as obesity, sleep apnea, heart failure, and diabetes mellitus.

Therefore, the pathophysiology of arrhythmias is complex, and inflammation likely plays a role in both the onset and duration of episodes. However, the role of pharmacotherapy aimed at inflammation and prevention of arrhythmias remains unclear, probably because AF is almost always a multifactorial disease and also because the target studied in preventing this inflammatory burden may also be present in the comorbidities of AF.
